# Impact of Angiotensin-Converting Enzyme Inhibitors (ACEIs) on the Efficacy of Immunotherapy in Metastatic NSCLC

**DOI:** 10.3390/medsci14040420

**Published:** 2026-07-23

**Authors:** Samer Abu-Rafe, Noa Shani Shrem, Abed Agbarya, Asmah Miari, Ronen Brenner, Yulia Dudnik, Ashraf Abu Jama, Sondos Shalata, Keren Rouvinov, Nashat Abu Yasin, Lama Tourkey, Adan Khalaily, Raya Bdair, Alexander Yakobson, Natalie Maimon Rabinovich, Walid Shalata

**Affiliations:** 1 Internal Medicine Department, Soroka Medical Center, Beer Sheva 84105, Israel; 2 Faculty of Health Sciences, Ben Gurion University of the Negev, Beer Sheva 84105, Israel; 3 The Legacy Heritage Cancer Center, Larry Norton Institute, Soroka Medical Center, Beer Sheva 84105, Israel; 4 Department of Oncology, Bnai Zion Medical Center, Haifa 31048, Israel; 5 Faculty of Medicine, Tel Aviv University, Tel Aviv 69978, Israel; 6 Department of Oncology, Meir Medical Center, Kfar Saba 44180, Israel; 7 Oncology Institute, Edith Wolfson Medical Center, Holon 58220, Israel; 8 Nutrition Unit, Galilee Medical Center, Nahariya 22000, Israel

**Keywords:** angiotensin-converting enzyme inhibitors (ACEIs), immunotherapy, non-small cell lung cancer, adenocarcinoma, squamous cell carcinoma, PD-L1, PD-1

## Abstract

Background: Immune checkpoint inhibitors (ICIs) have significantly improved outcomes in metastatic non-small cell lung cancer (NSCLC), yet only a subset of patients derives durable benefit, suggesting that host and tumor-related factors may modify treatment efficacy. The renin–angiotensin system has been implicated in regulation of the tumor microenvironment, and angiotensin-converting enzyme inhibitors (ACEIs) have therefore been proposed as potential modulators of immunotherapy response. Material and methods: We conducted a retrospective observational cohort study including patients with advanced metastatic NSCLC treated in the first-line setting with treatment-based immunotherapy, with or without chemotherapy, between January 2017 and September 2025. Chronic ACEI exposure was defined as continuous use for at least two years prior to initiation of immunotherapy. Progression-free survival (PFS) and overall survival (OS) were analyzed using Kaplan–Meier estimates and compared using the log-rank test, and multivariable Cox proportional hazards models were adjusted. Results: Among 446 eligible patients, 71 (16%) received ACEIs and 375 (84%) did not. The median age of the cohort was 67.5 years, and 70% were male. Adenocarcinoma was the predominant histology (67.7%), and most patients received chemo–immunotherapy (81.6%), while 18.4% received immunotherapy alone. PD-L1 expression ≥ 1% was present in 59.2% of patients. In the overall cohort, median PFS and OS were 12 and 15 months, respectively. Median OS was 17 months in the ACEI group compared with 14 months in the non-ACEI group (log-rank *p* < 0.047), while median PFS was 14 months versus 11 months, respectively (*p* = 0.066). The survival advantage was more pronounced for OS than for PFS and remained consistent after adjustment for clinical characteristics including age, sex, ECOG performance status, smoking status, histology, treatment regimen, and PD-L1 expression. Conclusions: These findings suggest that chronic ACE inhibitor use may be associated with improved outcomes in metastatic NSCLC patients treated with immune checkpoint inhibitors.

## 1. Introduction

Lung cancer remains one of the leading causes of cancer-related mortality worldwide. Approximately 85% of lung cancer cases are classified as non-small cell lung cancer (NSCLC), encompassing major histological subtypes such as adenocarcinoma, squamous cell carcinoma, and large cell carcinoma. Despite advances in early detection and therapeutic strategies, the prognosis of patients with advanced NSCLC remains poor [1,2].

Over the past decade, immunotherapy has emerged as a cornerstone in the management of non-small cell lung cancer, particularly through the use of immune checkpoint inhibitors (ICIs) targeting the PD-1 (Programmed Cell Death Protein 1) and PD-L1 (Programmed Cell Death Ligand 1) axis and Cytotoxic T-lymphocyte-associated protein 4 (CTLA-4) pathways [1,2,3]. These therapies have led to meaningful improvements in progression-free and overall survival in selected subsets of patients with NSCLC [1,2]. Nevertheless, clinical benefit from immunotherapy is not universal, and treatment responses vary considerably, with a substantial proportion of patients experiencing limited or no durable response [3,4].

Angiotensin-converting enzyme inhibitors are widely prescribed medications for the treatment of hypertension, heart failure, and other cardiovascular conditions. Beyond their cardiovascular effects, increasing evidence indicates that the renin–angiotensin system (RAS) plays a role in cancer biology, including tumor growth, angiogenesis, inflammation, and regulation of the tumor immune microenvironment [5]. Preclinical studies have demonstrated that inhibition of the RAS may reduce tumor-associated fibrosis, normalize tumor vasculature, and enhance infiltration of immune effector cells into the tumor microenvironment, thereby potentially improving antitumor immune responses and therapeutic efficacy [6].

Based on these immunomodulatory and anti-angiogenic effects, ACE inhibitors have been proposed as potential modifiers of immunotherapy outcomes. Observational clinical studies have suggested that concomitant use of RAS inhibitors, including ACEI, may be associated with improved survival outcomes in cancer patients treated with immune checkpoint inhibitors, although findings remain inconsistent and data specific to NSCLC are limited [7,8].

Because the interaction between ACE inhibitors and ICIs remains unclear, this study investigates the impact of ACE inhibitor therapy on the efficacy of immunotherapy in patients with metastatic NSCLC. Specifically, we compared PFS and OS between NSCLC patients treated with PD-1/PD-L1 inhibitors who were receiving ACE inhibitors and those who were not, to clarify the potential influence of ACE inhibitor use on immunotherapy outcomes.

## 2. Materials and Methods

### 2.1. Patient Enrollment

This retrospective, observational, non-interventional study included patients with wild-type metastatic NSCLC (absence of specific targetable genetic mutations such as EGFR, ALK, MET, RET or ROS1). The analysis comprised individuals treated in the first-line setting with either chemo–immunotherapy or ICI monotherapy, in accordance with routine clinical practice. ICI regimens included pembrolizumab or the combination of ipilimumab and nivolumab, administered with or without chemotherapy. The study period extended from January 2017 through September 2025, with follow-up data available until November 2025. Collected data encompassed patient demographics (including age at diagnosis and sex), treatment characteristics (type of ICI regimen and use of concomitant chemotherapy), and clinical outcomes, specifically OS and PFS. Additional recorded variables included PD-L1 expression status, dates of treatment initiation and discontinuation, sites of disease progression, and ECOG performance status. This multicenter retrospective study was approved by the Institutional Review Boards of Soroka University Medical Center (approval No. 0329; approved on 14 January 2025), Bnai Zion Medical Center (approval No. 0023-22-BNZ; approved on 30 January 2022), Meir Medical Center (approval No. 0108-23MMC; approved on 22 April 2025), and Edith Wolfson Medical Center (approval No. 0179-25-WOMC; approved on 3 December 2025). The study was conducted in accordance with the principles of the Declaration of Helsinki. Due to the retrospective nature of the study and the use of de-identified data, the requirement for informed consent was waived by the respective Institutional Review Boards.

### 2.2. Inclusion Criteria

Adult patients (≥18 years) with metastatic NSCLC were eligible for inclusion if they received ICIs, either as monotherapy or in combination with chemotherapy, during the study period. All included patients were required to have complete baseline demographic, clinical, pathological, and treatment-related data available. Chronic use of ACEIs was documented and defined as continuous administration for at least two years prior to the initiation of immunotherapy. To allow adequate assessment of treatment response and to minimize the confounding effect of rapidly progressive disease, only patients with a minimum PFS of four months were included. This criterion was applied to exclude individuals with highly aggressive disease and poor early prognosis, thereby optimizing follow-up duration and enabling a more reliable evaluation of the potential impact of ACEI use on outcomes associated with ICI therapy.

### 2.3. Exclusion Criteria

Patients were excluded from the analysis if they had incomplete or missing baseline clinical or demographic information, unclear documentation regarding immunotherapy regimen or ACEI exposure status, or insufficient follow-up data to allow for outcome evaluation. Individuals who discontinued treatment immediately after initiation or were lost to follow-up at an early stage were also excluded.

### 2.4. Statistical Analysis

Descriptive statistics were used to summarize the baseline demographic, clinical, and molecular characteristics of the patients. Continuous data with non-normal distributions were presented as medians (range), while categorical variables were reported as frequencies (percentages). Comparisons between patients receiving angiotensin-converting enzyme inhibitors and those not receiving ACEI were performed. PFS and OS were defined as the time from initiation of ICI to documented disease progression, death from any cause, or last follow-up, as applicable. Survival outcomes were estimated using the Kaplan–Meier method and compared between groups using the log-rank test. Multivariable Cox proportional hazards regression models were constructed to evaluate the association between ACEI exposure and survival outcomes while adjusting for potential confounders, including histological subtype, age, type of ICI, sex, smoking status, PD-L1 expression, and treatment regimen. Hazard ratios with corresponding 95% confidence intervals were reported.

All statistical tests were two-sided, and a *p*-value of less than 0.05 was considered statistically significant. Statistical analyses were performed using standard statistical software.

## 3. Results

Of 468 patients that were screened, the study cohort comprised 446 patients, of whom 71 (16%) received ACEI and 375 (84%) did not. The median age for the entire cohort was 67.5 years (range, 41–84). Patients treated with ACEI were slightly older, with a median age of 69.3 years (range, 53–83), compared with 67.2 years (range, 41–84) in the non-ACEI group.

Overall, 134 patients (30.0%) were female and 312 (70.0%) were male. In the ACEI group, 23 patients (32.4%) were female and 48 (67.6%) were male, while in the non-ACEI group, 111 patients (29.6%) were female and 264 (70.4%) were male. Regarding histology, adenocarcinoma was the predominant subtype, observed in 302 patients (67.7%), whereas 144 patients (32.3%) had squamous cell carcinoma. Among ACEI users, 51 (71.8%) had adenocarcinoma and 20 (28.2%) had squamous histology. In the non-ACEI group, 251 patients (66.9%) had adenocarcinoma and 124 (33.1%) had squamous cell carcinoma. In terms of smoking status, 64 patients (14.3%) were never smokers, 220 (49.3%) were current smokers, and 162 (36.3%) were former smokers. In the ACEI group, 11 patients (15.5%) were never smokers, 39 (54.9%) were current smokers, and 19 (26.8%) were former smokers. Among non-ACEI patients, 53 (14.1%) were never smokers, 181 (48.3%) were current smokers, and 143 (38.1%) were former smokers.

Performance status according to ECOG was 0 in 126 patients (28.3%), 1 in 241 (54.0%), and ≥2 in 79 (17.7%). In the ACEI group, ECOG 0, 1, and ≥2 were observed in 24 (33.8%), 36 (50.7%), and 11 (15.5%) patients, respectively. In the non-ACEI group, 101 patients (26.9%) had ECOG 0, 206 (54.9%) had ECOG 1, and 74 (19.7%) had ECOG ≥ 2.

Most patients received chemo–immunotherapy (364, 81.6%), while 82 (18.4%) received immunotherapy alone. Among ACEI users, 56 patients (78.9%) received chemo–immunotherapy and 15 (21.1%) received immunotherapy alone. In the non-ACEI group, 308 (82.1%) received chemo–immunotherapy and 67 (17.9%) received immunotherapy alone. Regarding the type of immunotherapy, 268 patients (60.1%) were treated with pembrolizumab and 180 (39.9%) with the combination of ipilimumab plus nivolumab. Pembrolizumab was more frequently administered in the ACEI group (52 patients, 73.2%) compared with the non-ACEI group (214 patients, 57.1%), whereas ipilimumab plus nivolumab was less common among ACEI users (19 patients, 26.8%) compared with non-users (161 patients, 42.9%). PD-L1 expression < 1% was observed in 182 patients (40.8%), while 264 (59.2%) had PD-L1 ≥ 1%. In the ACEI group, 33 patients (46.5%) had PD-L1 < 1% and 38 (53.5%) had PD-L1 ≥ 1%. In the non-ACEI group, 149 patients (39.7%) had PD-L1 < 1% and 226 (60.3%) had PD-L1 ≥ 1% (Table 1).

**Figure 1:**
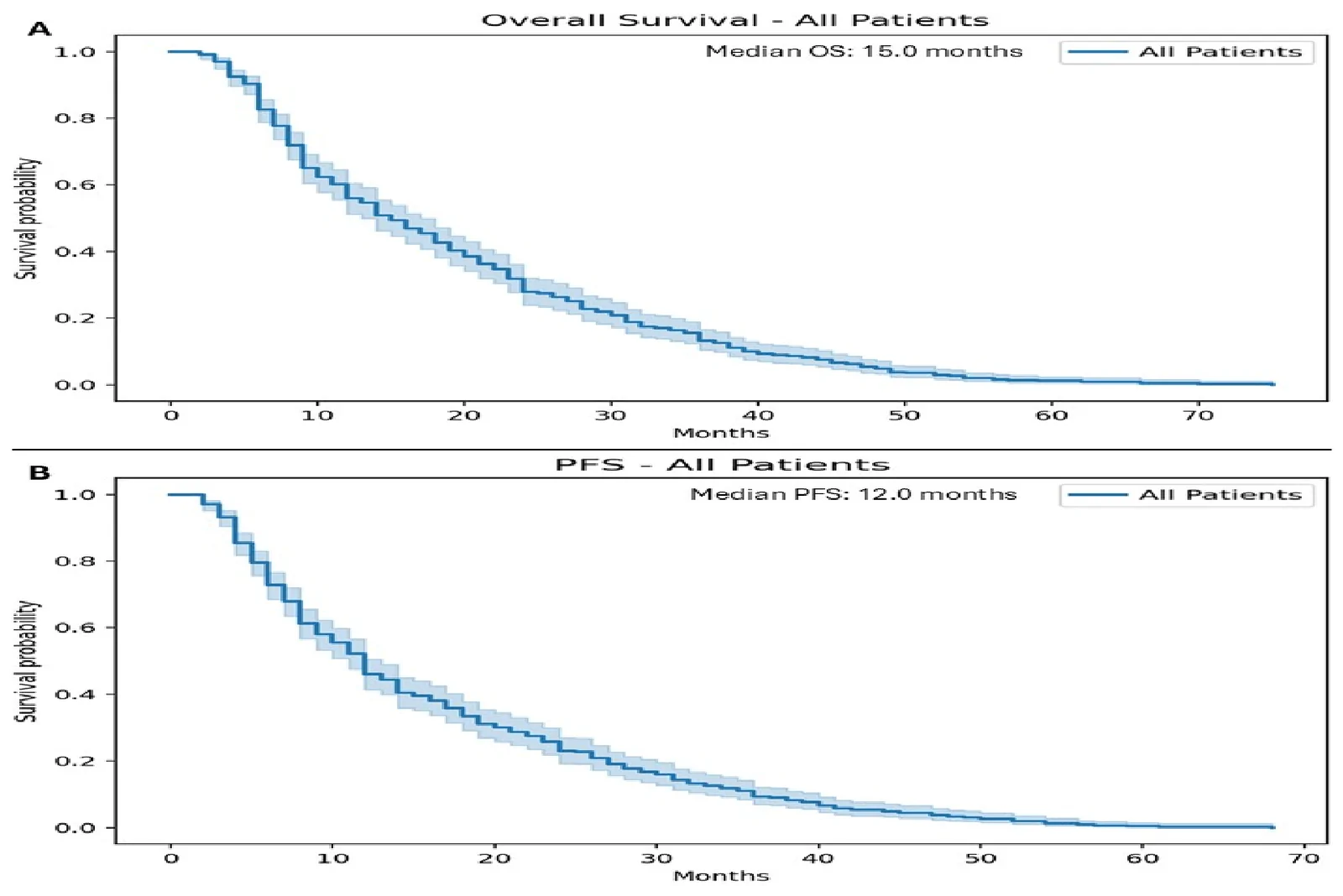
Kaplan–Meier survival curves for the overall study population illustrating overall survival (OS) and progression-free survival (PFS). Panel (**A**) presents median OS for all patients, and Panel (**B**) presents median PFS for all patients.

The entire cohort demonstrated a median OS of 15 months. Similarly, median PFS for the whole population was 12 months. Overall, the relatively close separation between median PFS and OS indicates a moderate post-progression survival interval, consistent with real-world metastatic NSCLC populations treated with immune checkpoint inhibitors (Figure 1).

**Figure 2:**
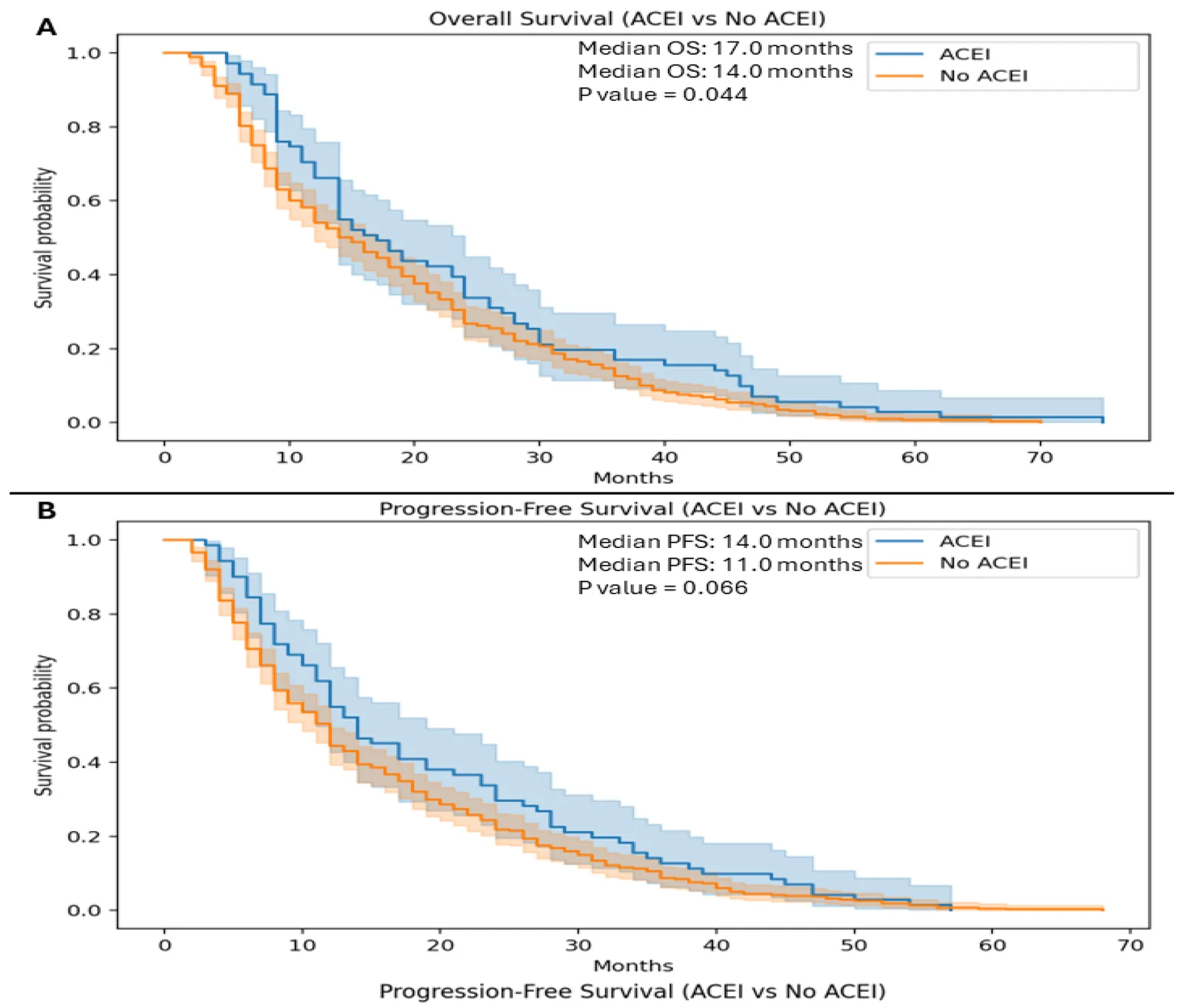
Kaplan–Meier estimates comparing survival outcomes between patients with chronic ACE inhibitor exposure and those without ACEI therapy. (**A**) Median OS was 17 months in the ACEI group compared with 14 months in the non-ACEI group. This difference was statistically significant according to the log-rank test (*p* = 0.044). (**B**) The Median PFS was 14 months among ACEI users versus 11 months among non-users; however, this difference did not reach statistical significance (log-rank *p* = 0.066).

The analysis of two cohorts comparing the patient group who were on chronic ACEI treatment to those without ACEI revealed distinct patterns in median OS and statistical significance. in terms of overall survival, the analysis demonstrated an improved OS among patients receiving ACE inhibitors compared with those not treated with ACE inhibitors. The median OS was 17 months in the ACEI group compared to 14 months in the non-ACEI group, with a statistically significant difference (log-rank *p* = 0.044). The survival curves separated early during treatment and remained consistently apart throughout follow-up, suggesting a sustained treatment-modifying effect rather than a late incidental divergence. For progression-free survival, patients treated with ACE inhibitors showed a numerically longer PFS (14 vs. 11 months), although this did not reach statistical significance (log-rank *p* = 0.066). Despite the lack of formal significance, the persistent separation of curves over time suggests a clinically meaningful trend favoring ACE inhibitor exposure (Figure 2).

**Figure 3:**
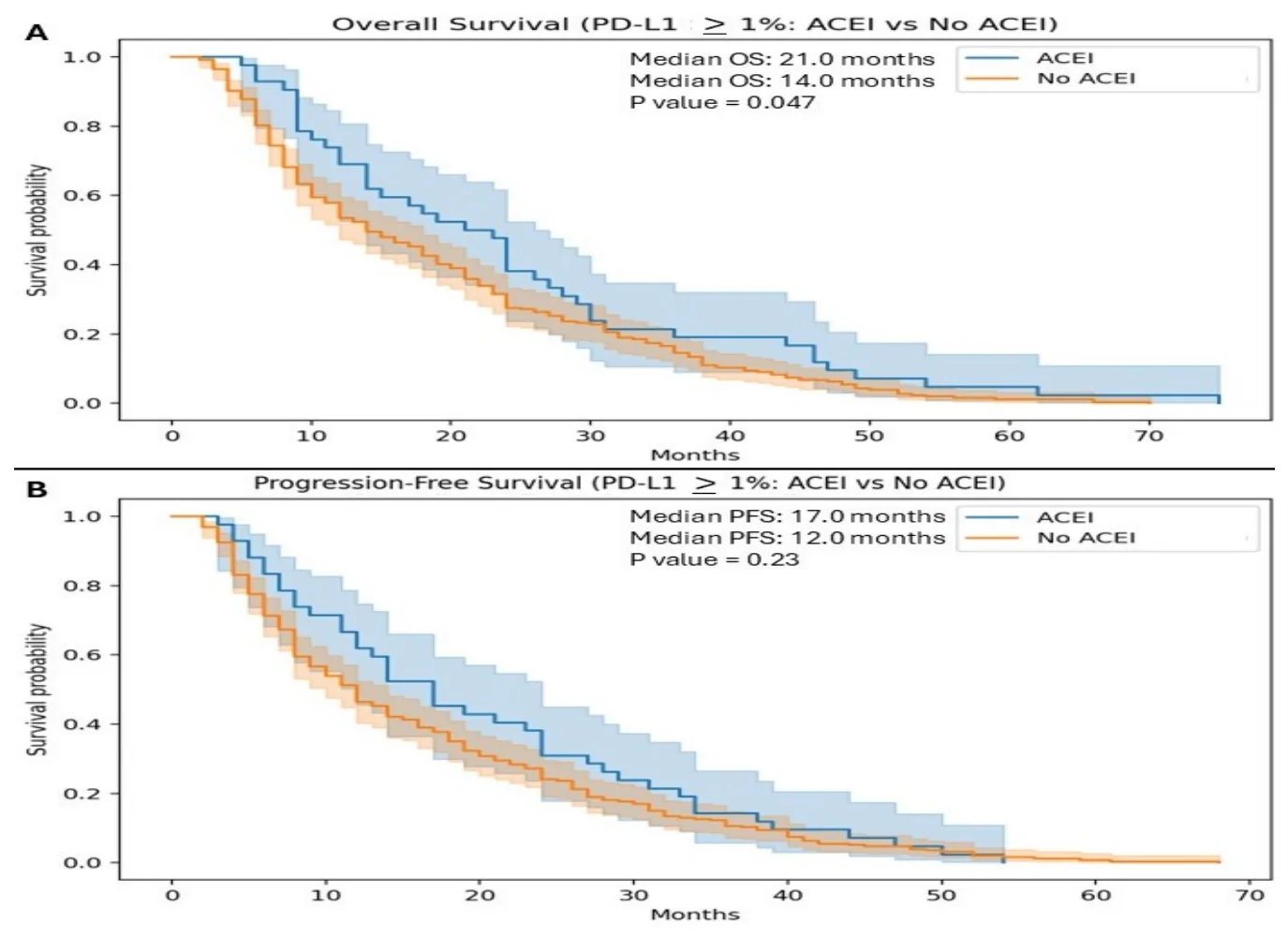
Kaplan–Meier survival analyses in the subgroup of patients with PD-L1 expression ≥ 1% comparing outcomes between chronic ACE inhibitor users and non-users are presented. (**A**) Patients receiving ACE inhibitors demonstrated a significantly prolonged median OS compared with those not exposed to ACE inhibitors (21 vs. 14 months, respectively; log-rank *p* = 0.047). The survival curves show a sustained separation over time, supporting a clinically meaningful survival advantage in this PD-L1–positive population. (**B**) The Median PFS was numerically longer in the ACEI group compared with the non-ACEI group (17.0 vs. 12.0 months); however, this difference did not reach statistical significance (log-rank *p* = 0.23).

Regarding the PD-L1-based subgroup analysis, it indicated a differential association between ACE inhibitor use and survival outcomes. Among patients with PD-L1 expression ≥ 1%, treatment with ACEI was associated with a significantly prolonged median overall survival compared with non-ACEI exposure (21 vs. 14 months, respectively), as confirmed by log-rank testing (*p* = 0.047). These findings suggest that the survival advantage observed with ACEI use may be more pronounced in patients with PD-L1-expressing tumors. In contrast, PFS demonstrated only a numerical improvement among patients receiving ACE inhibitors (17 vs. 12 months), without reaching statistical significance (log-rank *p* = 0.23). The observed divergence between overall survival and progression-free survival outcomes may indicate that ACE inhibition exerts its effect beyond initial radiographic disease control, potentially through modulation of post-progression tumor behavior, sustained immune microenvironment activity, or enhanced responsiveness to subsequent lines of therapy (Figure 3).

**Figure 4:**
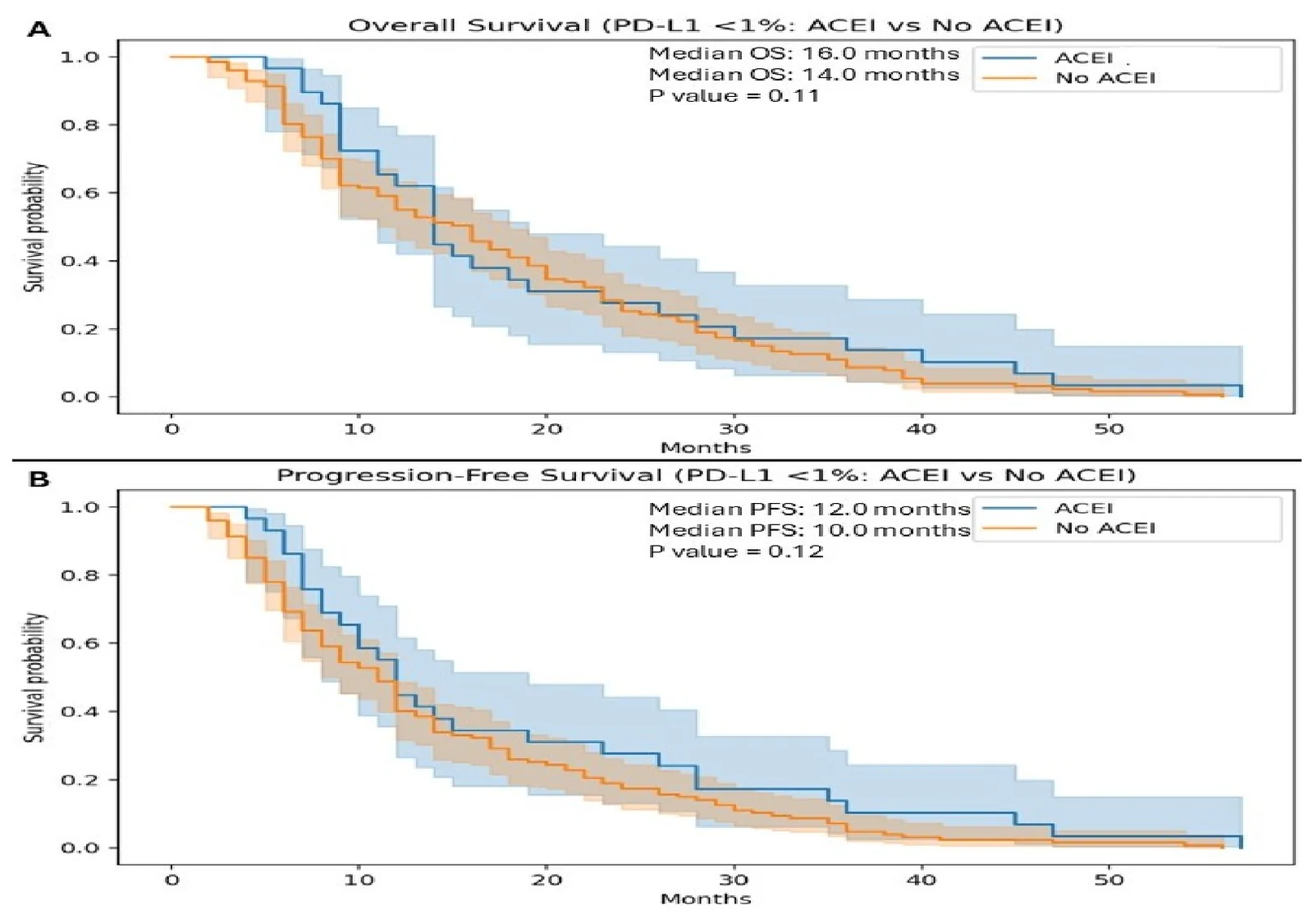
Kaplan–Meier survival analyses in patients with PD-L1 expression < 1% comparing outcomes between chronic ACE inhibitor users and non-users. (**A**) Median overall survival was 16 months in the ACEI group compared with 14 months in the non-ACEI group; however, this difference did not reach statistical significance (log-rank *p* = 0.11). The survival curves demonstrate limited and inconsistent separation over time, suggesting attenuation of the survival effect in tumors with low PD-L1 expression. (**B**) The Median progression-free survival was 12 months among ACEI users versus 10 months among non-users, without a statistically significant difference between groups (log-rank *p* = 0.12). The modest and non-sustained curve divergence further supports a limited impact of ACE inhibition in this subgroup. Shaded areas represent 95% confidence intervals.

Among patients with PD-L1 expression < 1%, exposure to ACE inhibitors was not associated with a statistically significant improvement in overall survival. Median OS was 16 months in the ACEI group compared with 14 months in the non-ACEI group (log-rank *p* = 0.11). Similarly, progression-free survival demonstrated only a modest numerical difference (12 vs. 10 months, respectively), which did not reach statistical significance (log-rank *p* = 0.12). Kaplan–Meier curves in the PD-L1 < 1% subgroup showed minimal and inconsistent separation between treatment cohorts, indicating a reduced or attenuated effect of ACE inhibition in tumors characterized by low baseline immune activation. Collectively, these findings suggest that the potential survival benefit associated with ACE inhibitor use may be context-dependent and more likely to manifest in the presence of a pre-existing immune-responsive tumor microenvironment (Figure 4).

**Figure 5:**
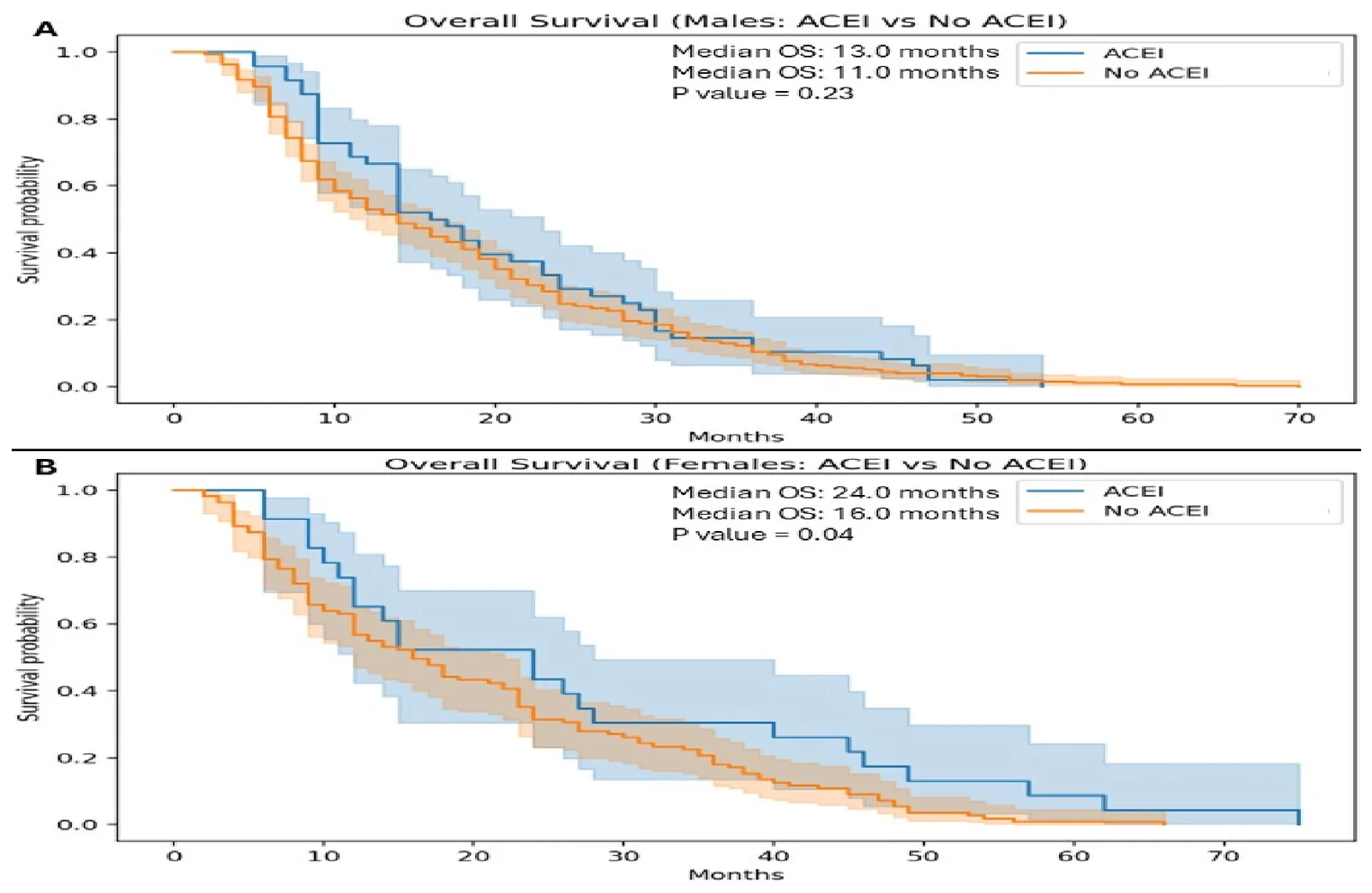
Kaplan–Meier overall survival analyses according to gender, comparing patients with chronic ACEI exposure to those without ACEI therapy. (**A**) Among males, ACE inhibitor use was not associated with a statistically significant improvement in overall survival. Median OS was 13 months in the ACEI group compared with 11 months in the non-ACEI group (log-rank *p* = 0.23). The survival curves demonstrate only modest and non-sustained separation between groups. (**B**) In contrast, among female patients, ACE inhibitor exposure was associated with a significant survival advantage. Median OS was 24 months in ACEI users versus 16 months in non-users (log-rank *p* = 0.04). The Kaplan–Meier curves in females show a more pronounced and sustained divergence over time. Collectively, these findings suggest a potential sex-specific interaction between ACE inhibition and antitumor immune response, with the observed survival benefit predominantly driven by female patients.

When analyzed according to gender, the association between chronic ACE inhibitor exposure and survival differed between male and female patients. Among male patients, chronic ACE inhibitor treatment was not associated with a statistically significant improvement in overall survival. Median OS was 13 months in ACEI users versus 11 months in non-users (log-rank *p* = 0.23). In contrast, among female patients, ACE inhibitor exposure was associated with a significant survival advantage. Median OS was 24 months in the ACEI group compared with 16 months in the non-ACEI group (log-rank *p* = 0.04). The more pronounced and sustained curve separation observed in females suggests a potential gender-specific interaction between the renin–angiotensin system and tumor immune microenvironment (Figure 5).

**Figure 6:**
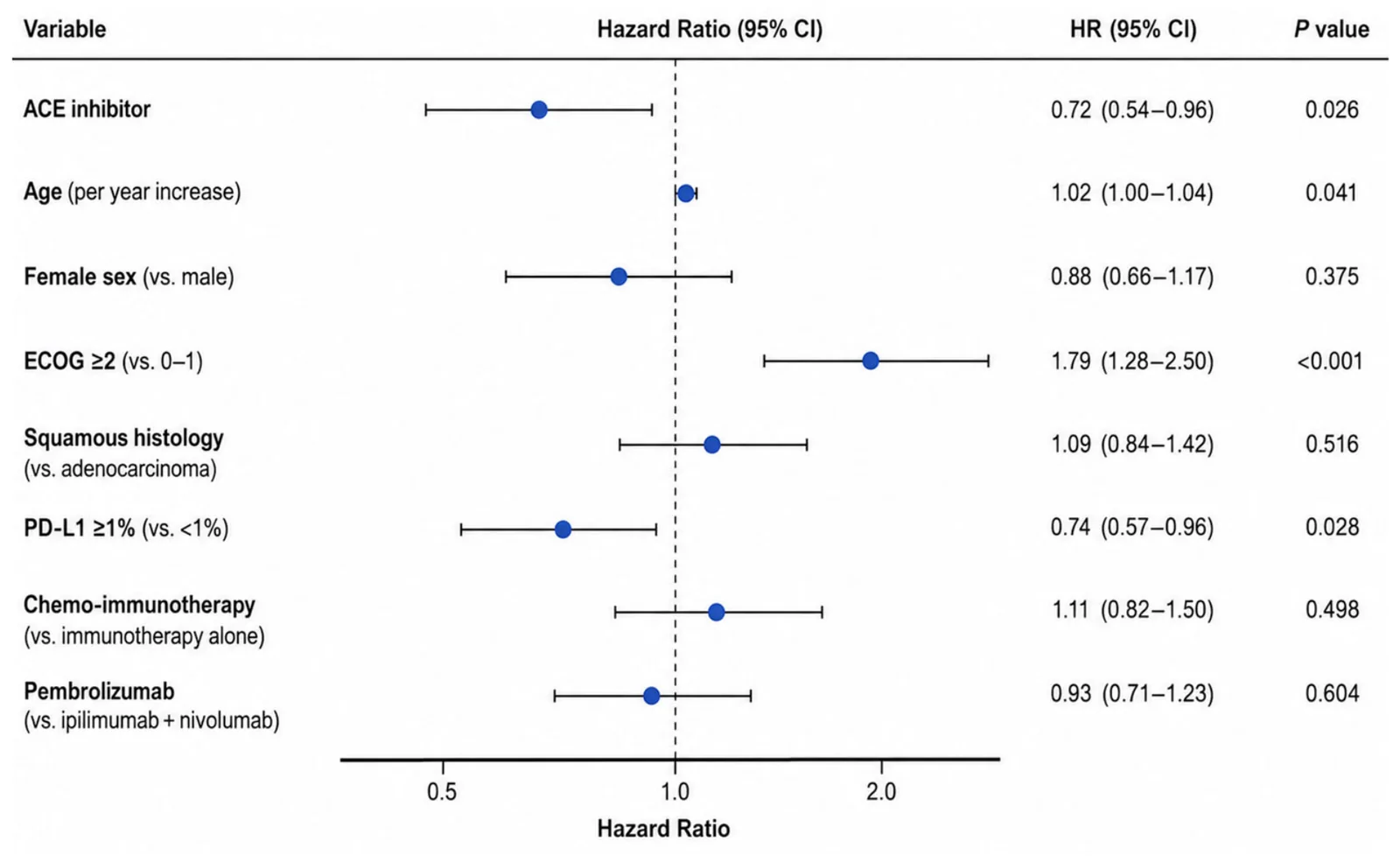
Multivariable Cox proportional hazards analysis for overall survival. Forest plot demonstrating the adjusted hazard ratios (HRs) and 95% confidence intervals (CIs) for baseline clinical and treatment-related variables associated with overall survival in patients with advanced NSCLC treated with immune checkpoint inhibitors. Chronic ACE inhibitor (ACEI) use remained independently associated with improved overall survival (HR 0.72, 95% CI 0.54–0.96, *p* = 0.026), corresponding to a 28% reduction in the risk of death after adjustment for age, sex, ECOG performance status, histology, PD-L1 expression, treatment regimen, and type of immunotherapy. ECOG performance status ≥ 2 was independently associated with significantly worse survival (HR 1.79, 95% CI 1.28–2.50, *p* < 0.001), whereas PD-L1 expression ≥ 1% was associated with improved survival (HR 0.74, 95% CI 0.57–0.96, *p* = 0.028). Increasing age was associated with a modest increase in mortality risk (HR 1.02 per year, 95% CI 1.00–1.04, *p* = 0.041). Female sex, squamous histology, treatment regimen (chemo–immunotherapy versus immunotherapy alone), and type of immune checkpoint inhibitor (pembrolizumab versus ipilimumab plus nivolumab) were not independently associated with overall survival after multivariable adjustment. The vertical dashed line represents the null effect (HR = 1.0); values to the left indicate reduced mortality risk, whereas values to the right indicate increased mortality risk.

To determine whether the association between chronic ACEI use and overall survival was independent of established prognostic factors, a multivariable Cox proportional hazards regression analysis was performed adjusting for age, sex, ECOG performance status, smoking status, histological subtype, PD-L1 expression, treatment regimen, and type of immunotherapy. After adjustment, chronic ACEI use remained independently associated with improved overall survival (adjusted HR 0.72, 95% CI 0.54–0.96, *p* = 0.026), corresponding to a 28% reduction in the risk of death. ECOG performance status ≥ 2 was independently associated with significantly inferior overall survival (adjusted HR 1.79, 95% CI 1.28–2.50, *p* < 0.001), while PD-L1 expression ≥ 1% was independently associated with improved survival (adjusted HR 0.74, 95% CI 0.57–0.96, *p* = 0.028). Increasing age was associated with a modest increase in mortality risk (adjusted HR 1.02 per year, 95% CI 1.00–1.04, *p* = 0.041). In contrast, sex, histological subtype, treatment regimen, and the type of immune checkpoint inhibitor were not independently associated with overall survival after multivariable adjustment (Figure 6).

## 4. Discussion

NSCLC represents about 80–85% of all lung cancer diagnoses and continues to be one of the main causes of cancer-related mortality globally [1,2]. Although treatment options have markedly progressed over the last decade, outcomes for metastatic disease remain poor, with wide variability in both therapeutic response and survival [2]. Despite substantial advances achieved with immune checkpoint inhibition, durable clinical benefit is restricted to a fraction of treated patients, reinforcing the need to clarify the biological and clinical variables that shape response to immunotherapy [3,4].

In this retrospective cohort study of patients with metastatic non-small cell lung cancer treated with immune checkpoint inhibitors, chronic exposure to angiotensin-converting enzyme inhibitors was associated with improved clinical outcomes. Patients receiving ACE inhibitors experienced significantly longer overall survival and a numerically longer progression-free survival compared with those not receiving these agents. These findings suggest that modulation of the renin–angiotensin system may influence the efficacy of immunotherapy in lung cancer and are consistent with a biologically plausible interaction. However, given the retrospective observational design, these findings demonstrate an association rather than causality and should therefore be interpreted cautiously [8,9,10,11].

Importantly, baseline clinical characteristics between the groups did not favor the ACEI cohort. Patients receiving ACE inhibitors were older and had a similar or slightly less favorable performance status distribution, factors that are typically associated with inferior oncologic outcomes. Despite this, survival was improved in the ACEI group, arguing against a simple selection bias. In addition, histologic distribution, smoking status, and PD-L1 expression were broadly comparable between groups, suggesting that the observed survival difference is unlikely to be explained by known major predictive biomarkers of immunotherapy response. The magnitude of overall survival benefit compared with the modest progression-free survival difference further supports a treatment-interaction effect rather than differences in disease biology at baseline.

The observed survival benefit is biologically plausible. Beyond their cardiovascular effects, renin–angiotensin system inhibitors are increasingly recognized as modulators of the tumor microenvironment [7]. Angiotensin II contributes to tumor fibrosis, abnormal angiogenesis, hypoxia, and recruitment of immunosuppressive cells [7,12,13]. ACE inhibition has been shown to normalize tumor vasculature, reduce stromal density, improve tissue perfusion, and enhance infiltration of cytotoxic T lymphocytes [8,14]. These microenvironmental changes may increase tumor accessibility to immune cells and thereby improve responsiveness to immune checkpoint blockade. These experimental observations provide a biologically plausible framework for interpreting our clinical findings. Nevertheless, whether these mechanisms directly account for the survival benefit observed in our cohort remains speculative, and mechanistic as well as prospective clinical studies are required to establish causality.

Interestingly, the survival advantage was more pronounced for overall survival than for progression-free survival. This pattern is often observed with immunomodulatory exposures and suggests that ACE inhibitors may not primarily increase initial response rates but instead improve the durability of immune response or post-progression disease control [15,16]. In other words, ACEI exposure may contribute to the generation of long-term responders rather than early radiologic responders. Such a phenomenon is consistent with the delayed and sustained clinical benefit characteristic of immunotherapy.

Previous studies evaluating renin–angiotensin system inhibition in patients receiving immune checkpoint inhibitors have yielded heterogeneous results. Some reports demonstrated improved survival in patients receiving ACE inhibitors or angiotensin receptor blockers during PD-1/PD-L1 blockade, whereas others showed neutral effects [10,17,18]. Differences in tumor types, treatment lines, exposure duration, and patient selection likely account for this variability [17].

Although the mechanism underlying the observed sex-specific association remains unclear, several biological hypotheses may be considered. Sex hormones, particularly estrogen, regulate the renin–angiotensin system by modulating the balance between the classical ACE/angiotensin II/AT1 receptor axis and the alternative ACE2/angiotensin-(1–7)/Mas receptor pathway, which has been associated with anti-inflammatory and immunomodulatory effects [19,20,21]. In parallel, sex-related differences in innate and adaptive immune responses, mediated partly by sex hormones and sex chromosome-related mechanisms, may influence the tumor immune microenvironment and response to immune checkpoint blockade. However, direct evidence linking sex-specific RAS regulation to differential ACEI efficacy during immune checkpoint inhibition is currently lacking. Moreover, the observed sex-specific association should be considered exploratory and hypothesis-generating. Because multiple subgroup analyses were performed, the possibility of chance findings, particularly in the female subgroup, cannot be excluded and requires confirmation in adequately powered prospective studies [19,20,21,22,23].

In lung cancer specifically, several retrospective analyses have suggested improved outcomes with concomitant RAS blockade, particularly when exposure preceded immunotherapy initiation, supporting a priming effect on the tumor microenvironment. Conversely, studies including mixed tumor populations or late initiation of ACEI therapy tended to show weaker associations, suggesting that timing of exposure may be critical. Our findings align with studies demonstrating improved survival in patients chronically exposed to ACEI prior to ICI initiation, reinforcing the hypothesis that long-term stromal remodeling rather than acute pharmacologic synergy underlies the clinical effect. The subgroup analyses should be interpreted cautiously. Although the observed associations across PD-L1 expression and sex are biologically interesting, the present study was not specifically powered for these comparisons. Furthermore, because multiple subgroup analyses were performed, the possibility of error cannot be excluded. Therefore, these findings should be regarded as exploratory and hypothesis-generating until validated in independent prospective cohorts [24]. The consistency of the signal across different checkpoint inhibitor regimens in our cohort further supports a mechanism independent of a specific drug and related instead to immune accessibility of the tumor.

Our study provides additional data specifically in first-line metastatic NSCLC, where the impact of concomitant medications on immunotherapy efficacy remains insufficiently characterized. Notably, patients receiving ACE inhibitors in our cohort were older, a factor usually associated with worse oncologic outcomes, yet they demonstrated improved survival. This observation strengthens the possibility of a true biologic interaction rather than a purely prognostic bias.

Several mechanisms may explain the association between ACE inhibitor exposure and improved outcomes. Angiotensin II promotes tumor hypoxia through abnormal angiogenesis [13], and hypoxic tumors are less responsive to immune checkpoint inhibitors [20]. ACE inhibition improves perfusion and oxygenation, potentially enhancing immune activity. Renin–angiotensin signaling also activates TGF-β pathways and promotes recruitment of myeloid-derived suppressor cells, both of which suppress antitumor immunity [25]; blocking this pathway may restore immune responsiveness. In addition, tumor fibrosis limits lymphocyte penetration, and ACE inhibition reduces stromal rigidity, allowing improved immune cell infiltration [8,25]. Collectively, preclinical studies suggest that these mechanisms may facilitate conversion of immune-excluded tumors into more immune-inflamed phenotypes, thereby potentially enhancing responsiveness to immune checkpoint inhibition.

The generally consistent trend across clinical subgroups is compatible with a systemic biologic interaction rather than a histology-specific phenomenon. However, these subgroup analyses should be interpreted cautiously because they were exploratory and not powered for definitive comparisons. The absence of a strong PFS difference alongside a significant OS benefit further argues against simple treatment selection bias and instead supports improved long-term immune control [15]. Clinically, ACE inhibitors are inexpensive and widely used medications. Our findings indicate that continuing ACE inhibitors during immunotherapy appears safe and may be beneficial. However, these results do not justify initiating ACE inhibitors solely for oncologic purposes outside prospective clinical trials [17,18].

This study has several limitations inherent to its retrospective design. Residual confounding by comorbidities such as hypertension and cardiovascular disease cannot be excluded. Indication bias and differences in medical follow-up patterns may also have influenced survival outcomes. Medication adherence and dosing were not systematically verified, and angiotensin receptor blockers were not analyzed separately. In addition, chronic ACEI exposure was defined as continuous use for at least two years before initiation of immune checkpoint inhibitor therapy. Although this definition was intended to identify sustained long-term exposure, the two-year cutoff is not based on a universally accepted standard and may have resulted in exposure misclassification. Furthermore, changes in ACEI therapy after initiation of immunotherapy, including treatment continuation, discontinuation, dose modification, or switching, were not consistently available because of the retrospective nature of the study. Consequently, ACEI exposure was assessed only at baseline and could not be analyzed as a time-varying variable. Additionally, patients with extremely aggressive disease were excluded because of the minimum progression-free survival requirement of four months. Although this criterion was intended to minimize inclusion of patients who discontinued treatment before adequate response assessment, it may have introduced selection bias by preferentially including patients with more favorable early clinical courses. Consequently, the generalizability of our findings to the broader population of patients with metastatic NSCLC receiving immune checkpoint inhibitors, particularly those with rapidly progressive disease, may be limited. Furthermore, this eligibility criterion may also have introduced immortal time (guarantee-time) bias because patients were required to remain progression-free long enough to satisfy the inclusion criteria [25]. Therefore, the magnitude of the observed association should be interpreted cautiously. Although multivariable adjustment was performed to account for measured confounders, residual confounding cannot be excluded, and causality cannot be established from this retrospective observational study. Prospective studies using predefined exposure assessment and time-dependent analytical approaches are needed to validate these findings.

## 5. Conclusions

Chronic ACE inhibitor use in patients with metastatic non-small cell lung cancer treated with immune checkpoint inhibitors was associated with improved overall survival and a trend toward longer progression-free survival. These findings support a potential interaction between the renin–angiotensin system and antitumor immunity. While causality remains unproven, ACE inhibitors may represent a clinically relevant modifier of immunotherapy response and warrant prospective investigation.

## Figures and Tables

**Table 1:** Demographics of participants in our cohort.

Characteristics	Overall (%, *N* = 446)	ACEI (*N* = 71, 16%)	No ACEI (*N* = 375, 84%)
**Age (years)**			
Median (range)	67.5 (41–84)	69.3 (53–83)	67.2 (41–84)
**Gender, *n* (%)**			
Female	134 (30.0)	23 (32.4)	111 (29.6)
Male	312 (70.0)	48 (67.6)	264 (70.4)
**Histology, *n* (%)**			
Adenocarcinoma	302 (67.7)	51 (71.8)	251 (66.9)
Squamous cell carcinoma	144 (32.3)	20 (28.2)	124 (33.1)
**Smoking status, *n* (%)**			
Never	64 (14.3)	11 (15.5)	53 (14.1)
Current	220 (49.3)	39 (54.9)	181 (48.3)
Past	162 (36.3)	19 (26.8)	143 (38.1)
**ECOG, *n* (%)**			
0	126 (28.3)	24 (33.8)	101 (26.9)
1	241 (54.0)	36 (50.7)	206 (54.9)
2+	79 (17.7)	11 (15.5)	74 (19.7)
**Type of Treatment, *n* (%)**			
Chemo–immunotherapy	364 (81.6)	56 (78.9)	308 (82.1)
Only immunotherapy	82 (18.4)	15 (21.1)	67 (17.9)
**Type of Immunotherapy, *n* (%)**			
Pembrolizumab	268 (60.1)	52 (73.2)	214 (57.1)
Ipilimumab + Nivolumab	180 (39.9)	19 (26.8)	161 (42.9)
**PD-L1 values, *n* (%)**			
PD-L1 < 1%	182 (40.8)	33 (46.5)	149 (39.7)
PD-L1 ≥ 1%	264 (59.2)	38 (53.5)	226 (60.3)

Abbreviation: *N*, number; ACEI, Angiotensin-converting enzyme inhibitors; PD-L1, Programmed Cell Death Ligand 1; ECOG, Eastern Cooperative Oncology Group.

## Data Availability

The original contributions presented in this study are included in the article. Further inquiries can be directed to the corresponding author.

## Abbreviations

The following abbreviations are used in this manuscript:

ACEI	Angiotensin-Converting Enzyme Inhibitor
CTLA-4	Cytotoxic T-Lymphocyte-Associated Protein 4
ECOG	Eastern Cooperative Oncology Group
ICI	Immune Checkpoint Inhibitor
NSCLC	Non-Small Cell Lung Cancer
OS	Overall Survival
PD-1	Programmed Cell Death Protein 1
PD-L1	Programmed Death-Ligand 1
PFS	Progression-Free Survival
RAS	Renin–Angiotensin System
TGF-β	Transforming Growth Factor Beta
HR	Hazard Ratios
CI	Confidence Intervals
